# The value of the phase angle of bioelectrical impedance analysis to predict malnutrition in hemodialysis patients

**DOI:** 10.3389/fneph.2025.1478367

**Published:** 2025-03-03

**Authors:** Qingxuan Xiao, Na Xie, Xinyang Xiang, Ting Cao, Yingye Xie, Xiang Liang, Xiaoyan Su

**Affiliations:** ^1^ Department of Nephrology, DongGuan Tungwah Hospital, Dongguan, China; ^2^ Dongguan Key Laboratory of Precise Prevention & Treatment of Chronic Kidney Disease and Complications, Dongguan, Guangdong, China

**Keywords:** maintenance hemodialysis, malnutrition, bioelectrical impedance analysis, phase angle, 7-point-SGA

## Abstract

**Objectives:**

To investigate the validity of bioelectrical impedance analysis (BIA)-derived phase angle (PhA) as a predictor of malnutrition in maintenance hemodialysis (MHD) patients.

**Methods:**

A single-center, cross-sectional study of 126 MHD patients was conducted. A diagnosis of malnutrition was based on the 7-point Subjective Global Assessment (7-p-SGA) criteria. A Bioelectrical Impedance Analyzer was used to determine the PhA, fat mass (FM), muscle mass, and extracellular water/total body water (ECW/TBW) ratio. Biochemical indices and anthropometric measurements were also assessed. Using 7-p-SGA criteria, the patients were categorized into two groups: well-nourished and malnourished. General patient characteristics and the PhA values were compared between the two groups. A correlation analysis examined the relationship between PhA and the nutritional index. Logistic regression models and receiver operating characteristic curve analyses were used to identify independent factors for predicting malnutrition and determining their respective cutoff values.

**Results:**

The malnourished group had a significantly lower PhA than the well-nourished group (5.19° (5.81°, 4.09°) vs 6.13° (6.80°, 5.49°), *P* < 0.001). The PhA correlated positively with body mass index (BMI), albumin (Alb), and handgrip strength (HGS) (*P* < 0.05). However, there were no significant associations between PhA and FM or triceps skinfold thickness (TSF) (P > 0.05). Multivariate logistic regression analysis revealed that PhA, Alb, and BMI were independent predictors of malnutrition. Of these, BMI was the strongest predictor [odds ratio (OR) = 0.68; *P* < 0.001]. PhA also served as a secondary predictor of malnutrition (OR = 0.588; *P* = 0.035). Receiver operating characteristic curve analysis indicated that a PhA threshold value of approximately 5.78° was optimal for predicting malnutrition.

**Conclusion:**

PhA is a straightforward and reliable predictor of malnutrition in MHD patients, with an optimal cut-off value of 5.78° identified for diagnosing this condition.

## 1 Introduction

Hemodialysis is the primary renal replacement treatment for end-stage renal disease (ESRD). In China, 844,265 patients are receiving maintenance hemodialysis (MHD) by the end of December 2022. According to ESPEN, malnutrition is defined as “a state resulting from lack of intake or uptake of nutrition that leads to altered body composition (decreased fat free mass) and body cell mass leading to diminished physical and mental function and impaired clinical outcome from disease. Malnutrition can result from starvation, disease or advanced ageing, alone or in combination” (2). Malnutrition is a major complication and the leading cause of death for this patient population (3). Thus, the timely recognition and diagnosis of malnutrition are critical for improving prognosis and reducing healthcare costs. The Kidney Disease Outcome Quality Initiative (KDOQI) clinical practice guideline recommend at least biannual routine nutritional screening for hemodialysis patients (1). The prevalence of malnutrition among MHD patients ranges from 9.2% to 81%, with a median of 43% and an interquartile range of 28%–56%. This variance is due to differences in geography and the methodology used for evaluation (4).

KDOQI recommends using the 7-p-SGA to assess malnutrition in stage 5 chronic kidney disease (CKD5) patients (recommendation level 1B) (1). However, this method requires patients to receive an extensive medical history and has subjective anthropometric measurements that limit its clinical utility. Thus, there is an urgent need to develop an objective and straightforward method to evaluate malnutrition in MHD patients.

Bioelectrical impedance analysis (BIA) is a simple, widely utilized, non-invasive, and inexpensive method for assessing body composition. An electric current is sent through the body and the voltage is measured to determine the resistance and reactance of various body compartments (5). The derived PhA is a parameter derived from BIA that determines the ratio of the reactance to resistance. PhA has shown promise in predicting malnutrition, protein-energy wasting (PEW), sarcopenia, and clinical prognosis in hemodialysis patients (5–8). However, the PhA reference range for predicting malnutrition in hemodialysis patients remains unclear due to study differences in patient race, methodology, and equipment. The coexistence of PEW and malnutrition indicates the presence of malnutrition, inflammation, and wasting in patients with CKD (9). While prior studies have identified PhA values of 4.6° and 4.95° as optimal cutoff values for PEW (7, 10), the reliability of using PhA to predict malnutrition in hemodialysis patients has faced scrutiny (11).

This study evaluated the nutritional status of MHD patients using the 7-p-SGA criteria as the gold standard. The findings were used to determine the predictive value of PhA in identifying malnutrition among MHD patients and the optimal PhA cutoff value, providing valuable insights into its clinical application.

## 2 Materials and methods

### 2.1 Patients

**Figure 1:**
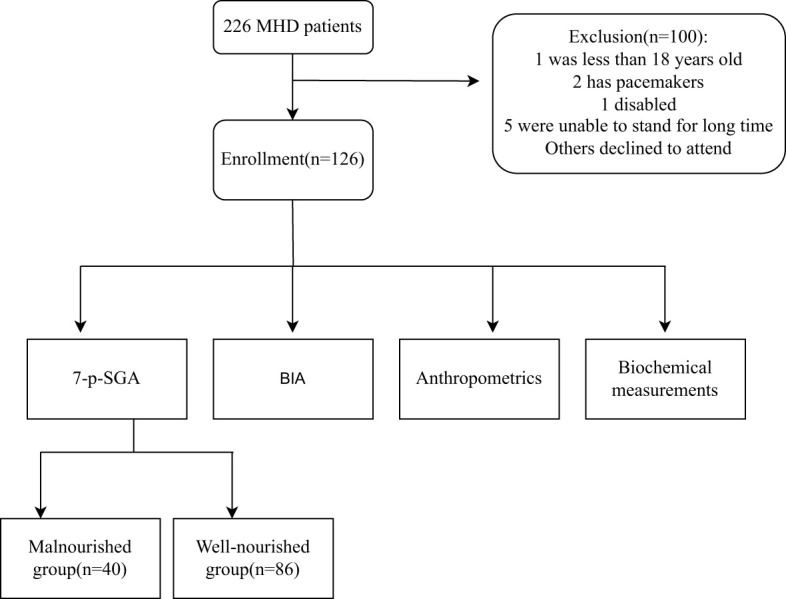
Study flow chart.

This observational study included 126 adult hemodialysis (HD) patients. Data was collected from June 2022 to October 2023 at DongGuan Tungwah Hospital, DongGuan, China. Ethical approval was obtained from the hospital’s research ethics committee, and all participants provided informed consent. Patients were included if they were aged ≥18 years, had undergone maintenance HD for >1 month, and had no contraindications for BIA, excluding pacemakers or limb deficiencies or inability to stand for extended periods. Individuals with active malignancy and recent hospital admissions within 1 month that potentially impacted their nutritional or functional status were excluded. A flow chart for this study is shown in **Figure 1**.

### 2.2 Diagnosis of malnutrition

Trained nurses conducted a 7-p-SGA questionnaire survey within 30 minutes post-dialysis. The survey included seven items assessing weight change, dietary intake, gastrointestinal symptoms, functional capacity, disease state relative to nutritional needs, and a physical examination encompassing loss of subcutaneous fat and muscle wasting. Each item was rated on a scale of 1 to 7, with higher scores indicating better nutrition. Patients were categorized as well-nourished (6–7 points), mild to moderately malnourished (3–5 points), or severely malnourished (1–2 points) (12). Given the limited number of patients in the severely malnourished group (n = 3), this study recategorized the patients into a well-nourished group and a malnourished group, with the latter including mild, moderate, and severe cases of malnutrition.

### 2.3 Anthropometrics and handgrip strength

Patient weight was measured using a Multi-frequency Body Composition Analyzer (TANITA, Japan), and height was recorded with a stadiometer. BMI was calculated as weight (kg) divided by height squared (m^2). Mid-arm circumference (MAC) was measured using a non-stretchable tape, and TSF was assessed with the Harpenden skinfold caliper. Mid-arm muscle circumference (MAMC) was calculated as MAMC (cm) = MAC (cm) – π × TSF (mm). HGS was measured using Jamar mechanical dynamometers (CAMRY, China). All measurements were conducted within 30 minutes post-dialysis. According to the 《Public Dietitian》, the normal reference values for MAMC are 24.8 cm in adult males and 21.0 cm in adult females, while those for TSF are 8.3 mm in adult males and 15.3 mm in adult females. The normal range of BMI for Chinese adults is 18.5-23.9 kg/m², with underweight defined as less than 18.5kg/m^2^, overweight as 24.0-27.9kg/m^2^, and obesity as 28.0kg/m^2^ or greater. According to the Asian Working Group for Sarcopenia (AWGS), the normal grip strength for adults is 28 kg for men and 18 kg for women (13).

### 2.4 Bioelectrical impedance analysis

Body composition was assessed by multifrequency BIA after 30 minutes of dialysis, as described previously (5). BIA-derived body components, including fat mass (FM), muscle mass, ECW, TBW, ECW/TBW, resistance (R), and reactance (Xc) (measured at 50 kHz), were recorded. PhA was computed using the formula: PhA (°) = arctangent (Xc/R) * 180/π.

### 2.5 Biochemical measurements

Blood samples were obtained before dialysis to determine serum urea, parathyroid hormone (PTH), Alb, triglycerides (TG), total cholesterol (TC), high-density lipoprotein-cholesterol (HDL-C), low-density lipoprotein cholesterol (VLDL-C), hemoglobin (Hb), total iron-binding capacity (TIBC), and high-sensitivity C-reactive protein (hs-CRP). Serum urea levels were also assessed after dialysis to calculate the urea Kt/V using the Daugirdas equation (14).

### 2.6 Statistical analysis

Statistical analyses were conducted using SPSS (version 20.0, SPSS Inc., Chicago, IL, USA). Patient demographic and clinical characteristics are presented as means ± standard deviations or median, interquartile ranges for continuous variables, and percentages for categorical variables. Comparisons between normally distributed, categorical, and non-normally distributed variables were made using the Student’s t-test, chi-square test, and Mann-Whitney test, respectively. The Spearman’s rank correlation coefficient was used to evaluate associations between PhA and nutritional indicators. Binary logistic regression was performed for multivariate analysis. Receiver operating characteristic curve analysis was conducted to analyze the ability of PhA to predict malnutrition using 7-p-SGA and the sensitivity and specificity were calculated. The area under the curve (AUC) denoted the test’s discriminative power. The concordance of the malnutrition diagnosis was assessed between PhA and 7-p-SGA using the Kappa value. Statistical significance was defined as a two-sided *P* value < 0.05.

## 3 Results

### 3.1 Basic characteristics of the well-nourished, and malnourished patients

The 126 HD patients had a mean age of 49.92 ± 15.09 and 65.1% were male. End-stage renal failure was caused by chronic glomerulonephritis (53/126; 42.1%), diabetes mellitus (29/126; 19%), and other diseases (49/126; 38.8%). The mean Kt/V was 1.33 ± 0.30. According to the 7-p-SGA criteria, 31.7% (40/126) of the patients were diagnosed as malnourished (three had severe malnutrition, 37 had mild or moderate malnutrition), while 68.2% (86/126) were well-nourished. Detailed characteristics of the patients in both groups are shown in **Table 1**. The median PhA of the well-nourished and malnourished groups were 6.13°(6.80°, 5.49°) and 5.19° (5.81°, 4.09°), respectively (*P* < 0.001). The malnourished group had a lower BMI, Alb, TSF, MAC, MAMC, muscle mass, and FM, and a higher PTH and Charlson’s disease index than the well-nourished group (all P < 0.05). HGS was slightly lower in the malnourished group, but this was not statistically significant (26.60 (20.45, 32.78) vs. 23.90 (16.28, 31.13), *P* = 0.065). No significant differences were observed in age, sex, primary disease, ECW/TCW, Hb, or hs-CRP between the two groups (*P* > 0.05).

**Table 1:** Patient characteristics and nutrition status of patients in the well-nourished and the malnourished groups.

Parameters	Total population	Well-nourished group	Malnourished group	95% confidence interval	*P*
Ages (years)	49.92 ± 15.09	48.47 ± 15.03	50.03 ± 14.92	−4.59 (−10.27–1.10)	0.113
Sex (n,%)				/	0.233
Male	82 (65.1%)	53 (61.6%)	29 (72.5%)		
Female	44 (34.9%)	33 (38.4%)	11 (27.5%)		
Original disease (n,%)				/	0.057
CGN	53 (42.1%)	41 (47.7%)	12 (30%)		
DKD	24 (19%)	12 (14%)	12 (30%)		
others	49 (38.8%)	33 (38.4%)	16 (40%)		
Dialysis vintage (days)	501 (236,948)	527 (288,958)	274 (186,935)	141 (−11–290)	0.075
CCI	3.3 (2,4)	3 (2,4)	4 (3,5)	−1 (−1–0)	0.004
KT-V	1.33 ± 0.30	1.32 ± 0.32	1.35 ± 0.25	–0.27 (−0.14–0.09)	0.641
BMI (Kg/m^2^)	22.30 ± 3.75	23.28 ± 3.67	20.02 ± 2.78	3.26 (0.59–2.09)	<0.001
Muscle mass (Kg)	43.66 ± 8.57	44.81 ± 8.53	41.35 ± 7.99	3.45 (0.29–6.62)	0.033
TSF (mm)	11.33 (7.33,15.83)	12.42 (8.13,16.33)	9.92 (6.33,14.48)	2.333 (0.333–4.433)	0.018
MAC (cm)	27.67 (25.40,30.33)	29.10 (26.83,31.23)	25.72 (22.48,27.53)	3.833 (2.367–5.333)	<0.001
MAMC (cm)	27.21 (25.12,29.93)	28.58 (26.50,30.82)	25.33 (22.32,27.22)	3.765 (2.35–5.168)	<0.001
HGS (Kg)	25.9 (19,32)	26.60 (20.45,32.78)	23.90 (16.28,31.13)	3.4 (−0.30–7.15)	0.065
FM (Kg)	13.75 (8.55,17.80)	15.60 (10.08,19.03)	10.45 (7.78,13.71)	4.725 (2.400–6.850)	<0.001
PHA	5.83 (6.64,5.21)	6.13 (6.80,5.49)	5.19 (5.81,4.09)	1.01 (0.58–1.47)	<0.001
ECW/TCW (%)	41.85 ± 3.64	52.93% ± 6.72%	55.21% ± 7.27%	−0.66 (−2.03–0.71)	0.087
Alb (g/L)	37.65 ± 4.32	38.33 ± 3.67	36.09 ± 5.10	2.25 (0.45–4.04)	0.015
Hb (g/L)	109.40 ± 17.82	108.70 ± 16.30	110.48 ± 20.75	−1.78 (−8.5–4.97)	0.635
PTH	193 (107,337)	181.00 (92.58,312.50)	245.00 (150.25,386.50)	−0.20 (−0.10–0.58)	0.027
Hs-CRP	1.42 (0.50,4.34)	1.27 (0.50,3.42)	1.87 (0.71,8.90)	–0.46 (−1.53–0.00)	0.067

CGN, Chronic glomerulonephritis; DKD, Diabetic nephropathy; CCI, Charlson comorbidity index; BMI, Body mass index; TSF, Triceps Skinfold; MAC, Mid-Arm Circumference; MAMC, Mid-Arm Muscle Circumference; HGS, Handgrip strength; FM, Fat Mass; PHA, Phase angle; ECW/TCW, Extracellular water/Total body water; Alb, Albumin; Hb, Hemoglobin; PTH, Parathyroid hormone; TG, triglycerides; HDL-C, High-density lipoprotein-cholesterol; Hs-CRP, High-sensitivity C-reactive protein.

### 3.2 Association between BIA indexes and nutrition-related indicators

PhA correlated positively with BMI, MAC, MAMC, muscle mass, Alb, lymphocyte counts, 7-p-SGA, HGS, and TC (all *P* < 0.05), and correlated negatively with age (r = −0.408, *P* = 0.001),ECW/TCW (r = −0.251, *P* = 0.005) and HDL-C (r = −0.251, *P* = 0.005) (**Table 2**). No significant associations were found between PhA and sex, TSF, fat mass, TG, VLDL-C, or TIBC (*P* > 0.05).

**Table 2:** Correlation between PhA and nutritional variables.

Parameters	R	*P*
Ages	-0.408	<0.001
Sex	0.055	0.529
BMI	0.244	0.006
TSF	0.122	0.175
MAC	0.287	0.001
MAMC	0.294	0.001
Fat mass	−0.31	0.730
Muscle mass	0.313	<0.001
ECW/TCW	−0.251	0.005
Alb	0.339	<0.001
Lymphocyte count	0.186	0.038
7-p-SGA	0.404	<0.001
HGS	0.386	<0.001
TIBC	0.890	0.319
VLDL-C	−0.050	0.586
HDL-C	−0.250	0.005
TC	0.236	0.008
TG	−0.06	0.517

BMI, Body mass index; TSF, triceps skinfold; MAC, Mid-arm circumference; MAMC, Mid-arm muscle circumference; ECW/TCW, extracellular water/total body water; Alb, Albumin; 7-p-SGA,7-point Subjective Global Assessment; HGS, Handgrip strength; TIBC, Total Iron-Binding Capacity; VLDL-C, Low-Density Lipoprotein Cholesterol; HDL-C, High-density Lipoprotein-Cholesterol; TC, Total Cholesterol; TG, Triglycerides.

### 3.3 Diagnostic value of PhA for malnutrition

After adjusting for other nutritional indicators, PhA (OR = 0.59, CI 0.36–0.96, *P* = 0.035) and BMI (OR = 0.68, CI 0.56–0.83, *P* < 0.001) were identified as predictive factors for malnutrition (**Table 3**). PhA had a slightly weaker predictive power than BMI.

**Table 3:** Logistic regression analysis of the predictive variables related to malnutrition in hemodialysis patients.

Parameters	b	OR	95%CI	*P*
PhA (°)	−0.531	0.588	0.359–0.963	0.035
ALB (g/L)	−0.123	0.884	0.778–1.004	0.058
Age (years)	0.025	1.025	0.983–1.069	0.251
BMI	−0.386	0.680	0.554–0.834	0.000
HGS	0.050	1.051	0.986–1.121	0.127

PhA, Phase angle; Alb, Albumin; BMI, Body mass index; HGS, Handgrip strength.

**Figure 2:**
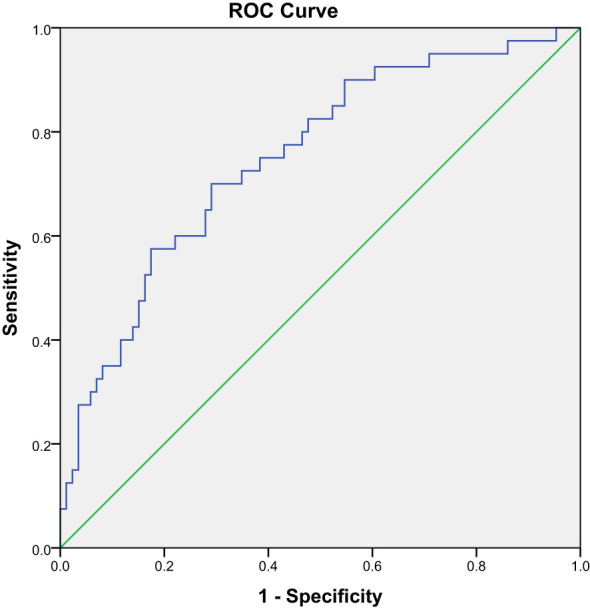
Receiver operating characteristic curve of the sensitivities and specificities of using PhA to predict malnutrition.

The optimal cutoff value for malnutrition was 5.78°, with an AUC of 0.75 (CI 0.659–0.842, *P* < 0.001) (**Figure 2**). Using this criterion, 42.1% (53/126) of patients were diagnosed as malnourished, with 70.0% sensitivity and 70.9% specificity. Concordance evaluation yielded a concordance rate of 90.5% and a Kappa value of 0.377 (*P* < 0.001) (**Table 4**).

**Table 4:** Malnutrition diagnosis using PhA and 7-p-SGA.

PhA ≤ 5.78°	7p-SGA		Total (n,%)
	Well-nourished group (n,%)	Malnourished group (n,%)	
Well-nourished group (n,%)	61 (70.9%)	25 (29.1%)	86 (100%)
Malnutrition group (n,%)	12 (30%)	28 (70%)	40 (100%)
Total (n,%)	73 (57.9%)	53 (42.1%)	126 (100%)
Kappa = 0.377 (P < 0.001).			

## 4 Discussion

PhA reflects cell membrane integrity, with larger angles indicating better cell function (15). While PhA has been used in the nutritional and functional assessment of cancer patients (16), its application in evaluating malnutrition in hemodialysis patients requires further investigation (17). The current study assessed the relationship between PhA and malnutrition in hemodialysis patients and affirmed the reliability of using PhA in this context.

In 2010, Oliveira et al. showed that PhA could serve as a reliable assessment tool for malnutrition in dialysis patients (18). However, this study only evaluated the correlation between PhA and nutritional indicators and did not conduct a multivariate regression analysis or determine the PhA threshold value. In 2016, researchers identified PhA as a more robust predictor of malnutrition in MHD patients than other BIA parameters (19). Han et al. also demonstrated that PhA is a reliable marker of nutritional status in non-dialysis CKD5 and peritoneal dialysis patients (20). However, additional validation has been proposed to confirm the utility of PhA in evaluating malnutrition associated with MHD (17). Inadequate concordance between PhA and SGA in MHD patients has also been reported (21). The current study validates the overall coherence of PhA in MHD patients using the 7-p-SGA criteria. The findings suggest that PhA could be a predictive tool for identifying MHD-associated malnutrition.

MHD patients were categorized into malnourished and well-nourished groups using the 7-p-SGA criteria. The malnourished group had a lower PhA than the well-nourished group [(5.19° (5.81°, 4.09°) vs. 6.13° (6.80°, 5.49°), *P* < 0.001]. Similarly, Rimsevicius et al. demonstrated that the 15^th^ and 25^th^ PhA percentiles in MHD patients corresponded to severe and mild malnutrition, respectively (19). Previous studies of chronic kidney disease patients showed that malnourished individuals have lower phase angles than well-nourished individuals (22). Since BIA requires measurements to be taken once fluids are redistributed after 30 minutes of dialysis, most patients declined participation in this study due to the prolonged waiting period. Some malnourished patients were also unable to participate in the body composition analysis because they could not endure extended periods of standing. Thus, only three severely malnourished patients were included in this study. As a result, only two groups were compared, the first consisting of patients with mild to severe malnutrition and the other with well-nourished individuals. Prior studies categorized patients into similar cohorts based on SGA assessment (21).

The current study identified a strong correlation between PhA and most nutritional indicators. Anthropometric measurements, including body mass index (BMI), serum albumin levels, and grip strength, serve as reliable indicators of nutritional status (1). Our study further confirmed that PhA exhibited a strong correlation with these indices. Consistent with our findings, other scholars have also reported a positive correlation between PhA and MAMC, MAC, and ALB (18). However, our study did not identify a significant association between PHA and either TSF or fat mass. Some scholars have found no significant correlation between PhA and TSF (18). We analyzed the possible reasons: firstly, there is inherent subjectivity in the measurement of TSF, and secondly, the observed effects may be attributable to the limited sample size. For fat mass assessment, several studies have indicated that BIA may not provide accurate evaluations (23). There were no significant differences in TC, TG, VLDL-C, and HDL-C between the sarcopenia and non-sarcopenia groups (7). Our study revealed that PhA was associated with TC and HDL-C but not with TG or VLDL-C. Therefore, further research is warranted to elucidate the relationship between blood lipids and nutrition. Low TIBC was associated with malnutrition (24); however, no significant correlation was found between PHA and TIBC in our study. Similarly, in a previous study on chronic periodontitis, TIBC was not found to be associated with malnutrition (25). As TIBC is a key laboratory indicator for diagnosing iron metabolism disorders, we analyzed the possible reasons for this discrepancy: First, it may be related to the patients’ iron supplementation regimens. Second, the lack of association might be due to the relatively small sample size. Advanced age is a well-established risk factor for malnutrition, and our study confirms that PhA exhibits an inverse relationship with age. Recent studies have confirmed that ECW/TCW serves as a marker of malnutrition in hemodialysis patients (26). Our study further reveals an inverse correlation between PhA and ECW/TCW. Previous studies have confirmed that PhA is strongly correlated with both the quadriceps femoris muscle and subcutaneous adipose tissue, as assessed using ultrasound guidance (27). PHA is significantly correlated with these nutritional parameters, thereby making it a valuable indicator for assessing malnutrition in dialysis patients.

Binary logistic regression analysis defined PhA as an independent predictor of malnutrition (OR = 0.588, 95% CI (0.359–0.963), P = 0.035). BMI was the strongest predictor of malnutrition in this study, in contrast to the findings of Laurynas et al., which found that BMI could not predict malnutrition. It should be noted, however, that the average BMI was higher in the Laurynas et al. study (25.93 ± 5.59) than in the current study (22.30 ± 3.75). This may be related to differences in the population and sample size, with the Laurynas et al. study having a smaller sample size and a higher proportion of severely malnourished patients. BMI also exhibits a higher degree of predictability for malnutrition than PhA (28). However, BMI is influenced by both body volume and adipose tissue content, changes that have minimal effect on PhA (18). Thus, when utilizing BMI to predict malnutrition, it is important to consider the impact of both body volume and adipose tissue content.

PhA cutoff values vary across studies and are influenced by multiple factors, including the manufacturing company and patient characteristics. Karavetian et al. determined that the PhA cutoff for malnutrition in MHD patients is ≤ 5.7°, consistent with the current study (29). Wang et al. found that the PhA cutoff value for sarcopenia in MHD patients was 4.95° (7) and Ding et al. confirmed a PHA cutoff value of 4.67° in MHD patients with sarcopenia (30). Tan et al. defined a PhA cutoff value for PEW in MHD patients of 4.6° (10). Segall et al. showed that a PhA of <6° was significantly associated with an increased risk of death in MHD patients (31). Bae et al. found that a PhA of < 4° was associated with worse clinical outcomes in MHD patients (8). The current study identified a PhA cutoff of 5.78°, with an AUC of 0.75 (CI 0.659–0.842, P < 0.001), a sensitivity of 70.0%, and a specificity of 70.9%. Despite the variation, a Kappa value of 0.377 (P < 0.001) suggests that these results were consistent. The PhA value in this study was high, likely because few patients with severe malnutrition were included. In addition, the diagnostic criteria for malnutrition, sarcopenia, and PEW differed. The term “sarcopenia” refers to a condition characterized by severe malnutrition. Additional studies that include more patients with severe malnutrition are needed to identify a PhA cut-off value for this population.

The current study has some limitations. First, because this was a single-center and small-scale study, causality could not be established. Second, the absence of severely malnourished patients may have inflated the PhA cutoff value. Third, the impact of changes in nutritional status on PhA was not explored.

## 5 Conclusion

The current study found that malnourished patients had significantly lower PhA levels, with an optimal cutoff value of 5.78°. These findings suggest that PhA could serve as a simple and reliable indicator for predicting malnutrition in hemodialysis patients. Future multicenter studies with larger sample sizes are needed to ascertain the optimal PhA cutoff. Longitudinal studies should also be conducted to explore the dynamic changes in PhA by nutritional status.

## Data Availability

The original contributions presented in the study are included in the article/supplementary material. Further inquiries can be directed to the corresponding authors.
